# Rapid and Quantitative Prediction of Tea Pigments Content During the Rolling of Black Tea by Multi-Source Information Fusion and System Analysis Methods

**DOI:** 10.3390/foods14162829

**Published:** 2025-08-15

**Authors:** Hanting Zou, Ranyang Li, Xuan Xuan, Yongwen Jiang, Haibo Yuan, Ting An

**Affiliations:** State Key Laboratory of Tea Plant Germplasm Innovation and Resource Utilization, Tea Research Institute, Chinese Academy of Agricultural Sciences, Hangzhou 310008, China; 13500436167@163.com (H.Z.); 13529036009@163.com (R.L.); 18334363220@163.com (X.X.); jiangyw@tricaas.com (Y.J.)

**Keywords:** black tea, tea pigments, machine vision, near-infrared spectroscopy

## Abstract

Efficient and convenient intelligent online detection methods can provide important technical support for the standardization of processing flow in the tea industry. Hence, this study focuses on the key chemical indicators—tea pigments in the rolling process of black tea as the research object, and uses multi-source information fusion methods to predict the changes of tea pigments content. Firstly, the tea pigments content of the samples under different rolling time series of black tea is determined by system analysis methods. Secondly, the spectra and images of the corresponding samples under different rolling time series are simultaneously obtained through the portable near-infrared spectrometer and the machine vision system. Then, by extracting the principal components of the image feature information and screening characteristic wavelengths from the spectral information, low-level and middle-level data fusion strategies are chosen to effectively integrate sensor data from different sources. At last, the linear (PLSR) and nonlinear (SVR and LSSVR) models are established respectively based on the different characteristic data information. The research results show that the LSSVR based on middle-level data fusion strategy have the best effect. In the prediction results of theaflavins, thearubigins, and theabrownins, the correlation coefficients of the testing sets are all greater than 0.98, and the relative percentage deviations are all greater than 5. The complementary fusion of the spectrum and image information effectively compensates for the problems of information redundancy and feature missing in the quantitative analysis of tea pigments content using the single-modal data information.

## 1. Introduction

As a kind of widely concerned teas, black tea has the typical quality characteristics of red soup, red leaves, and full taste [1]. Relevant studies have shown that the formation of these unique qualities is related to the characteristic active ingredients such as theaflavins (TFs), thearubigins (TRs), and theabrownins (TBs) generated during the production and processing [2,3,4]. Rolling is the key process that shapes these characteristics of black tea, and it is also the important process for the rapid generation and accumulation of TFs, TRs, and TBs. The primary purposes of this processing stage are to rupture tea cells, facilitate enzymatic oxidation reactions, and lay the material foundation for the subsequent fermentation process [5]. Real-time monitoring of tea pigments changes during the rolling process helps to accurately control the degree of rolling, thereby achieving standardized control of the processing. This has important practical significance for ensuring the stability of tea quality. At present, the quantitative analysis of tea pigments content in black tea rolled leaves is mainly based on system analysis methods [6,7]. The method determines the total content of TFs, TRs, and TBs by using the UV-visible spectrophotometer. Besides, the major monomer components of TFs can be quantitatively analyzed by the high-performance liquid chromatography (HPLC) [8]. These chemical analysis and detection techniques based on laboratory conditions take a long time for sample pretreatment and require specific experimental environments. In conclusion, in actual production, there is still a lack of the efficient and accurate detection methods. Based on above situation, this study aims to establish the efficient and intelligent quantitative evaluation methods for the changes in tea pigments content during the rolling of black tea.

Near-infrared spectroscopy (NIRS) technology is a widely used non-destructive testing method, which has the advantages of fast, accurate, and high cost-effectiveness [9,10]. NIRS can record the frequency doubling and combined frequency vibration absorption of hydrogen-containing groups in the molecular structure of substances. These transitions in vibration energy levels are characteristic of different chemical groups, which can produce absorption peaks at specific wavelengths. Based on this, through machine learning algorithms, spectral information can be correlated with the component content of the target substance. As another non-destructive testing method that can be used for quantitative analysis of chemical substances, machine vision (MV) can capture the surface color features of the sample to be tested through the high-resolution camera [11,12]. The color change on the sample surface caused by the generation and content variation of the target substance can be quantified by extracting the color models of the corresponding image of the sample through MV. Similarly, the relationship between image color information and the content of target substances can also be established through machine learning algorithms. NIRS can respond to the hydrogen-containing groups in the molecular structures of TFs, TRs, and TBs. And due to their generation and transformation, the appearance color of the rolled leaves also changes accordingly. It can be seen from this that NIRS and MV can be used for the quantitative analysis and research of tea pigments content during the rolling of black tea. To sum up, NIRS and MV respectively perceive the content changes of TFs, TRs, and TBs through the vibration information of tea pigments molecules and the surface color features of rolled leaves. Moreover, the data fusion methods can combine the sensor information of NIRS and MV, which can perceive the changes in the content of the target substance from multiple angles, obtain more feature information [13], and solve the problem of insufficient prediction accuracy existing in single-modal information.

**Figure 1:**
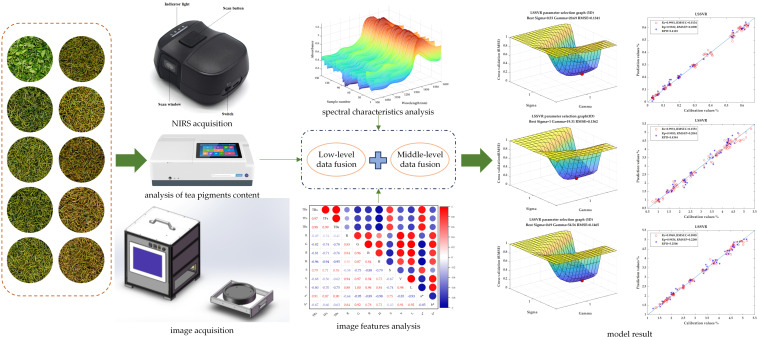
Flow.

In this study, the total content of TFs, TRs, and TBs in the samples is determined respectively by the system analysis methods. The portable near-infrared spectrometer is used to collect the spectral characteristic information of the corresponding samples. Meanwhile, the surface color features of the corresponding samples are captured by using the MV system. Subsequently, the spectral preprocessing methods are used to perform scattering correction, noise removal, baseline correction, and size scaling on the raw spectra. The characteristic extraction is carried out on the preprocessed spectra to capture the characteristic bands related to the content of the target substance. For image information, the changes in the color features are quantified by extracting RGB, HSV, and La*b* color models. In addition, standardize the feature variables in the above color models and extract the principal components. On this basis, through the principles of data information fusion [14], the prediction models of TFs, TRs, and TBs are established respectively based on low-level and middle-level data fusion. The selections of data information processing and data fusion methods are verified through PLSR, SVR, and LSSVR. And the model results are compared with the prediction model results based on single-mode data information. Ultimately, the best models for predicting the content of TFs, TRs, and TBs during black tea rolling are established. This has played a positive role in promoting the standardization of tea processing. The experimental flow of this study is shown in Figure 1.

## 2. Materials and Methods

### 2.1. Sample Preparation

The fresh tea leaves (one bud with two leaves) of Fuding Dabai were picked in Shengzhou, Zhejiang Province, China. After the fresh leaves were picked, the moisture content range of fresh tea leaves was measured to be 77.06–78.94% by the rapid moisture analyzer (MA35M-000230V1, Sartorious, Göttingen, Germany). First, spread the tea leaves evenly in the withering trough. During the withering process, the ambient temperature range is approximately 24–26 °C, and the ambient relative humidity range is approximately 60–62%. The withering process lasted approximately 11 h. After the withering, the moisture content of the withered leaves was measured to range from 60.66–61.56%. And, the withered leaves were evenly divided into different batches according to their mass for black tea rolling experiment.

The selected rolling machines models are 6CR-45 (Xiangfeng Machinery Co., Ltd., Tongxiang, China). The time and pressure setting principles for the rolling refer to NY/T 3222-2018 “Processing Technology Specifications for Gongou Black Tea”. In order to collect the different degrees of rolling samples, the specific settings are as follows: no-pressure for 20 min, light-pressure for 20 min, heavy-pressure for 10 min, light-pressure for 10 min, heavy-pressure for 10 min, light-pressure for 10 min, and light-pressure for 10 min. Sampling of rolled leaves starts from the end of withering. The samples are collected every 10 min throughout the rolling process, with 15 groups of samples collected each time. A total of 150 groups of rolling samples were collected for sensor information collection and quantitative analysis of tea pigments content.

### 2.2. Tea Pigments Content Quantitative Analysis

The total content of tea pigments (TFs, TRs, and TBs) was quantitatively analyzed using the methods of Zou et al. [11]. The contents of TFs, TRs, and TBs in each group of black tea rolled leaves samples were measured for three times in parallel.

### 2.3. Image and NIRS Information Acquisition

The MV system adopted in this study is an industrial all-in-one machine composed of core components such as industrial cameras, industrial lenses, shadowless light sources, control panels, and visual inspection software [11]. The system is a 360° shadowless dome light source using white LEDs, ensuring shadow-free and highly uniform illumination. The industrial camera (MV-CS020-10GMGC, Hikvision, Hangzhou, China) adopted has a high-resolution pixel of 20 million. The mechanical body of the entire machine is an aluminum frame structure. During the image acquisition process, each group of samples (of identical mass) was evenly spread out within the shooting range of the MV equipment. To reduce the impact of the surface morphology of the samples on subsequent data analysis, three different images were taken for each group of samples. This will be used to calculate the average color feature parameters in each group of sample images, thereby enhancing the representativeness of the data.

The portable NIR spectrometer used was model NIR-S-G1 (Innospectrum, Taiwan, China). Its spectral measurement range covers 900–1700 nm, with a resolution of 10–12 nm, the wavelength accuracy of ±1–2 nm, and a signal-to-noise ratio of 6000:1. The instrument exhibits excellent stability and accuracy. To ensure the stable operation of the instrument, the instrument was preheated for 30 min before data acquisition. Subsequently, whiteboard correction is performed. During the experiment, each group of samples (of identical mass) was evenly placed on the experimental platform, and three random scans were performed on different areas of the sample surface. The average of the three scans was used as the representative spectral data for the group.

Finally, the representative spectra and average color feature parameters are used as the inputs of the prediction models, while the parallel determination results of tea pigment content serve as the outputs. Establishing a one-to-one correspondence between the sensor data and the tea pigment content is essential for data analysis.

### 2.4. Data Set Partitioning

It is necessary to scientifically partition the training and testing sets prior to establishing prediction models in order to ensure the validity and reliability of data analysis. The training set ensures the sufficiency of model parameter learning, while the independence of the testing set can objectively reflect the generalization ability of the model. The division ratio of training and testing sets will also directly affect the final performance of the prediction model. Therefore, this study employs stratified random sampling to divide the data set into training set (70%) and testing set (30%), which not only ensures the full training of the models, but also ensures the reliability of the evaluation results. In summary, there are 105 training groups and 45 testing groups.

### 2.5. Image Feature Information Preprocessing

First of all, Pearson correlation analysis is adopted to quantify the strength of linear correlation between image color features and tea pigments. By revealing potential relationships between image color features and tea pigment content, this study aims to identify features that are highly correlated with the target variables. In addition, the extracted color feature variables are derived from three different color models (RGB, HSV, and La*b*), and there are differences in the calculation methods among these variables. So as to solve the modeling deviations caused by different units or numerical ranges of multi-source data, this study applies data standardization (Z-score) to normalize the data scales. This is conducive to ensuring the stability and comparability during model training. In the meantime, Based on the correlation analysis results, principal component analysis (PCA) is applied to eliminate redundant features and retain the essential information, thereby improving the computational efficiency [15,16].

### 2.6. Spectral Characteristic Information Preprocessing

The data of NIRS usually contain interference factors such as noise, light scattering, light intensity differences, and baseline drift during the acquisition process [17]. Spectral preprocessing can eliminate the interference of these non-target signals and enhance the effective information [18]. Besides, the full-band spectral data contain some wavelengths variables that are not related to the chemical composition to be determined [19]. The spectral characteristic wavelengths extraction algorithms are the effective methods for identifying the spectral characteristic most relevant to the substance being tested, and can further minimize redundancy and noise interference. In this study, seven spectral preprocessing methods and four spectral characteristic wavelength selection algorithms are adopted for comparative data analysis and validation.

Standard normal variate (SNV) is used to solve the light scattering problem caused by the uneven surface of the samples [20]. Multiplicative scatter correction (MSC), Savitzky-Golay algorithm with 1st order polynomial (S-G-1st), and Savitzky-Golay algorithm with 2nd order polynomial (S-G-2nd) are used to remove baseline drift caused by samples scattering and optical path difference [21]. In addition, Detrend is employed to eliminate low-frequency background interference [22]. Max-min normalization (Max-min) [23] is used to reduce light intensity differences to highlight the characteristic absorption peaks of the substances to be determined. Savitzky-Golay (SG) is used to eliminate high-frequency noise and suppress irrelevant signals [24].

The related studies have shown that the full-band spectrum will significantly increase the modeling time, and only the selected wavelengths are needed in practical applications [25]. In this study, bootstrapping soft shrinkage (BOSS) [26], competitive adaptive reweighted sampling (CARS) [27], interval variable iterative space shrinkage approach (i-VISSA) [28], and model adaptive space shrinkage (MASS) [29] are used to screen the spectral characteristic information.

### 2.7. Data Information Fusion Strategy

The data fusion methods generally adopt three approaches: low-level data fusion, middle-level data fusion, and high-level data fusion [14]. Low-level data fusion refers to the direct concatenation of raw vectors from different sources to form an integrated input matrix for predictive modeling. Middle-level data fusion refers to the process of extracting features from each data source separately before concatenating them into an integrated matrix for modeling. High-level data fusion is more complex, requiring not only feature extraction from different data sources but also the construction of separate multivariate models for analysis. Based on this, the outputs of these models are integrated for comprehensive decision-making. Currently, high-level data fusion has not been widely adopted due to its potential information loss. Given the amount of data to be integrated in this study is relatively small, the analysis is primarily conducted using low- level and mid-level data fusion strategies.

### 2.8. Establishment of Prediction Models

Partial least squares regression (PLSR) is a statistical modeling method used to solve linear prediction problems [30]. PLSR is especially suitable for data with high dimension, strong collinearity or limited sample size. It can establish the robust relationship between the predictor variable (X) and the response variable (Y) by projecting high-dimensional data into a low-dimensional space. In the researches of quantitative analysis of tea substances using spectra, PLSR has been widely applied.

Support Vector Regression (SVR) is an extension of the support vector machine (SVM) in regression problems. Its core idea is to construct the optimal regression hyperplane in the high-dimensional feature space through the kernel function and the principle of structural risk minimization. SVR is particularly suitable for nonlinear regression prediction problems [31]. The kernel function selected in this study is the radial basis kernel function (RBF). When using RBF, it is necessary to select the appropriate key parameters the c (penalty coefficient) and g (bandwidth parameter). The parameter (c) is utilized to balance the trade-off between training errors and model complexity, while (g) can be employed to regulate the decision boundary of the model. In this study, the c and g are optimized through grid search with 5-fold cross-validation (5-fold CV).

Least Squares Support Vector Regression (LSSVR) is the deformation algorithm of SVR [32]. LSSVR transforms the inequality constraint of standard SVR into equality constraint and solves it by least square loss function, which has higher computational efficiency. Similarly, RBF is used as the kernel function of LSSVR in this study. In regression prediction analysis of the LSSVR model, the kernel parameter (sigma) and regularization parameter (gamma) need to be adjusted to optimize the model results. Gamma is used to balance the model complexity and training error, and sigma affects the ability of the model to capture the nonlinear relationship of data. In the same way, grid search with 5-fold CV are used to choice the optimal parameter combination.

### 2.9. Evaluation Indexes of Prediction Models

The evaluation indexes of the prediction model selected in this study are: the correlation coefficient of calibration (training) set (Rc), the correlation coefficient of prediction (testing) set (Rp), root-mean-square error of calibration (training) set (RMSEC), root-mean-square error of prediction (testing) set (RMSEP), and residual prediction (testing) deviation (RPD) [33]. Rc and Rp values can express the strength of the relationship between the predicted values and the true values [21]. RMSEC and RMSEP values are used to express the deviation between the predicted values and the true values of the model [34], which helps to intuitively reflect the accuracy of the prediction. RPD determines the reliability and applicability of the model by comparing the prediction error of the model with the variability of the data itself.

## 3. Results and Discussion

### 3.1. Results Analysis of Tea Pigments Content

**Figure 2:**
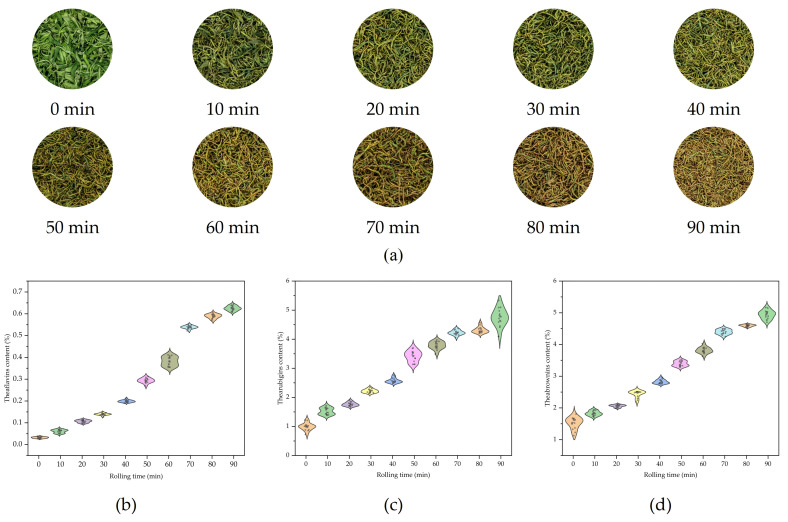
(**a**) Representative images of samples under different rolling time series; (**b**) changes in TFs content; (**c**) changes in TRs content; (**d**) changes in TBs content.

TFs is an important component of the flavor intensity and freshness of black tea, and it is also the oxidation products of catechins. TRs, as the main color-forming substance of black tea, is a secondary metabolite converted from TFs by oxidative polymerization. TBs is related to the astringency of black tea, and the accumulation of its content is a key factor causing the color of rolled leaves to darken. The representative images of samples under different rolling time series are shown in Figure 2a. The dynamic changes in content of TFs, TRs, and TBs during the rolling of black tea are shown in Figure 2b–d. The results show that during this period, the content of TFs, TRs, and TBs all show an upward trend with the increase of rolling time. Rolling disrupts the structure of tea leaf cells by mechanical force, facilitating the contact between enzymes and substrates, and triggering the oxidative polymerization of polyphenols. This indicates that the disruption of tea leaf cellular structures and the occurrence of oxidative polymerization reactions are the key factors for the generation and accumulation of tea pigments during rolling. The gradual increase in the content of these pigments substances also induced changes in the color features of the appearance of the rolled leaves.

### 3.2. Image Color Features Responses Analysis and Features Extraction

**Figure 3:**
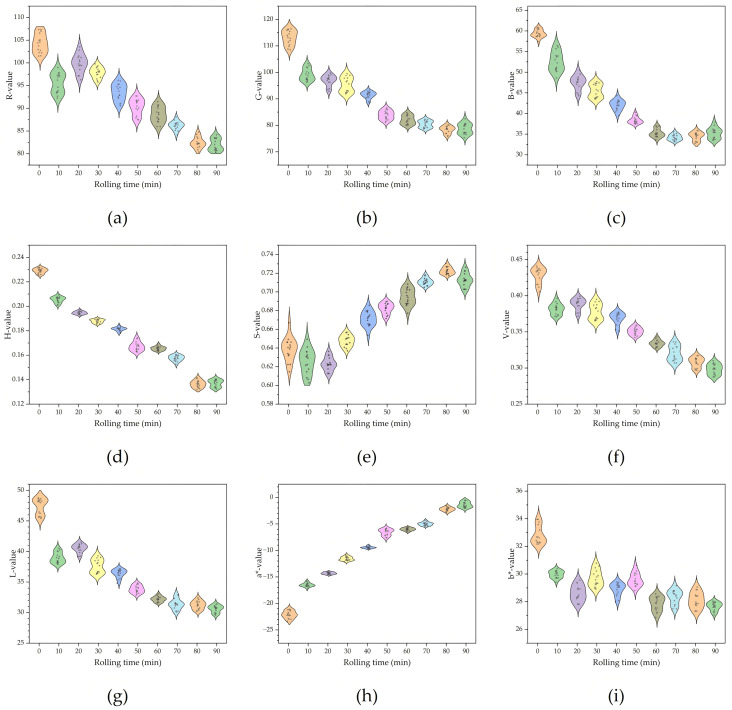
Image feature variables: (**a**) R, (**b**) G, (**c**) B, (**d**) H, (**e**) S, (**f**) V, (**g**) L, (**h**) a*, (**i**) b*.

In this study, the average color features variables extracted from the images include: R (red), G (green), B (blue), H (hue), S (saturation), V (brightness), L (brightness), a* (red-green degree), and b* (yellow-blue degree). The changes of color feature variables with rolling time are shown in Figure 3a–i. From the perspective of color feature parameters, the S and a* values show an upward trend with the increase of rolling time. The values of R, G, B, H, V, and L show a downward trend with the increase of rolling time. The results show that the synergistic variation of each color feature parameters under different rolling time correspond to the macroscopic phenomenon of the gradual deepening of the color of rolled leaves. The surface color changes of the rolled leaves can be quantitatively described through the above feature information.

**Figure 4:**
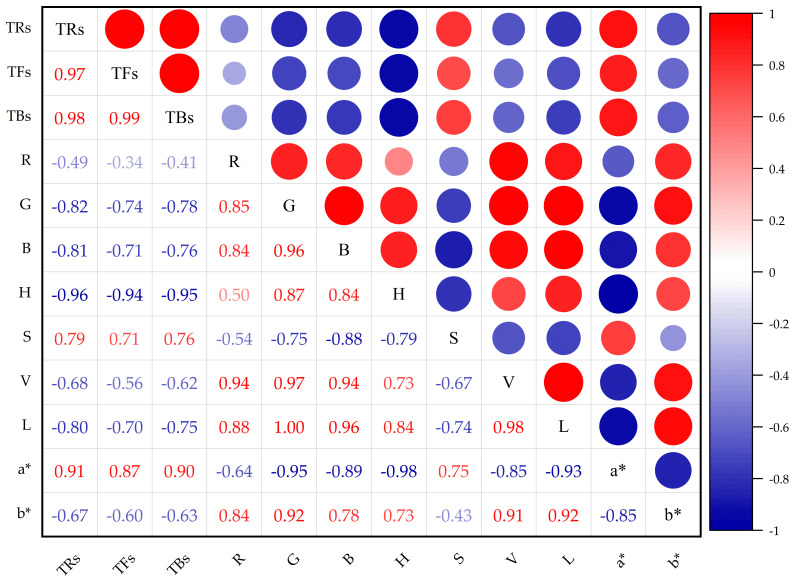
The results of Pearson correlation analysis.

Based on the above, the correlation analysis is conducted between the color feature variables and the corresponding tea pigments content. The analysis results are shown in Figure 4. Here, the red represents the positive correlation and the blue represents the negative correlation. Positive numbers represent the intensity of positive correlation, while negative numbers represent the intensity of negative correlation. The results show that for the content values of TFs, TRs, and TBs, different color feature variables have different positive/negative correlations with them. Besides, the response intensities of different color feature quantities show differences, and they also have varying degrees of correlation with each other. Therefore, after standardizing the data using Z-score, the possible collinearity among its variables is eliminated through PCA. The explanatory variances of the four principal components extracted are 70.50%, 13.29%, 7.71%, and 5.64% respectively. It indicates that while reducing the dimension of the data, the main information is retained by PCA.

### 3.3. Results of Prediction Models Based on Image Information

The results of the PLSR models constructed based on image color feature information are shown in Table 1. The data results show that, compared with only using Z-score as the pretreatment method, the combined use of Z-score + PCA can effectively improve the predictability of the models. This demonstrates the necessity and effectiveness of PCA in extracting features from image data. Based on the above analysis, this study uses Z-score + PCA as the image information pretreatment method, and applies SVR and LSSVR for modeling analysis to further verify and explore the effects of different methods. The results of the SVR and LSSVR models constructed based on image color feature information are shown in Table 2. It can be seen that after using the nonlinear models, the prediction results of the contents of TFs, TRs, and TBs have been significantly improved and enhanced. By contrast, the LSSVR model based on Z-score + PCA has the better prediction effect. However, machine vision technology can only capture the physical phenomenon of color change on the surface of the sample. Although the detection method has the advantages of intuitive and simple, in practical applications, the detection results are easily disturbed by factors such as the shooting environment. Therefore, multi-dimensional information perception technology should be developed to make up for the limitations of a single technology.

### 3.4. Spectral Characteristic Responses Analysis

**Figure 5:**
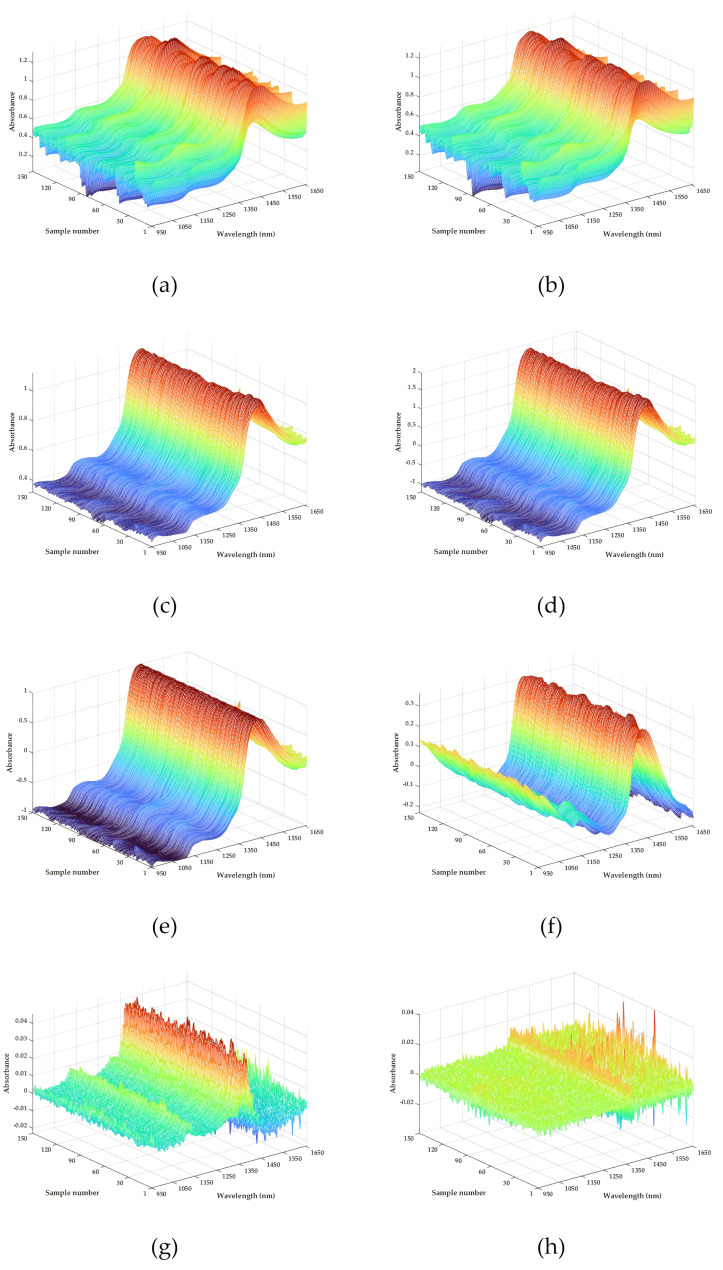
Raw spectrum and spectral pretreatment methods: (**a**) raw spectrum; (**b**) SG pretreatment; (**c**) MSC pretreatment; (**d**) SNV pretreatment; (**e**) Max-min pretreatment; (**f**) Detrend pretreatment; (**g**) S-G-1st pretreatment; (**h**) S-G-2nd pretreatment.

The raw spectrum of the rolled leaves is shown in Figure 5a. It can be seen that the spectrum shows obvious absorption peaks at 1170 and 1450 nm. The peak at 1170 nm may be derived from the joint frequency absorption band of C-H and O-H groups. The absorption peak at 1450 nm may be related to first-level frequency-doubled tensile vibration of the O-H group. The spectrum after pretreatments by SG, MSC, SNV, Max-min, Detrend, S-G-1st, and S-G-2nd are respectively presented in Figure 5b–h. The results show that the spectrum after SG pretreatment is the closest to the raw spectrum. However, the spectrum processed by Detrend, MSC, SNV, and Min-max have undergone significant changes, manifested as the contraction of spectral bands and the enhancement of absorption and reflection peaks. Compared with the above results, the spectrum changes after S-G-1st and S-G-2nd pretreatments are more significant, but these methods will also highlight the noise problem existing in the spectrum.

### 3.5. Results of Prediction Models Based on Spectral Characteristic

Firstly, the prediction models of PLSR are constructed based on raw spectrum and preprocessed spectrum. The results of the PLSR models established based on spectral characteristic information are shown in Table 3. The data results show that these pretreatment methods are helpful to improve the prediction effect on TFs, TRs, and TBs. Meanwhile, the pretreatment methods that performs best in PLSR models results are selected for further analysis and discussion. The results of the SVR and LSSVR models based on spectral characteristics are shown in Table 4. Comparative results indicate that the SNV-LSSVR, MSC-LSSVR, and SG-LSSVR models achieve the better prediction accuracy for TFs, TRs, and TBs, respectively.

**Figure 6:**
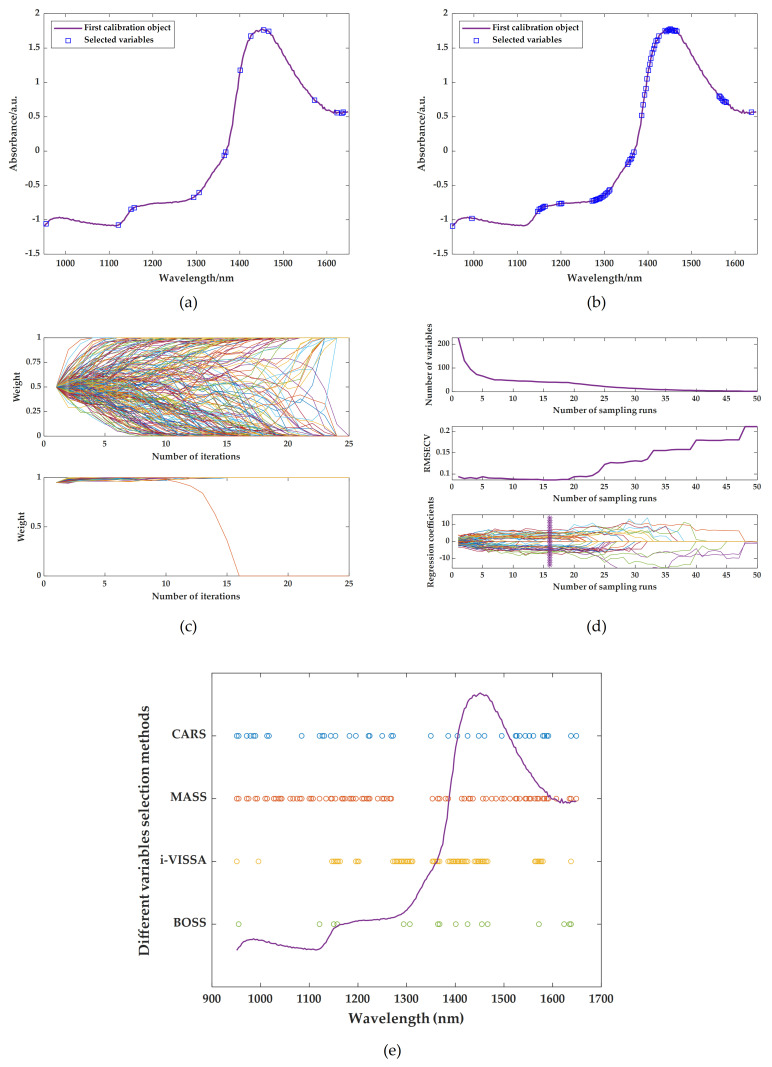
Optimal characteristic wavelengths selected by different variable screening methods: (**a**) extraction results of BOSS after SNV; (**b**) extraction results of i-VISSA after SNV; (**c**) extraction results of MASS after SNV; (**d**) extraction results of CARS after SNV; (**e**) comparison of characteristic wavelength results.

**Figure 7:**
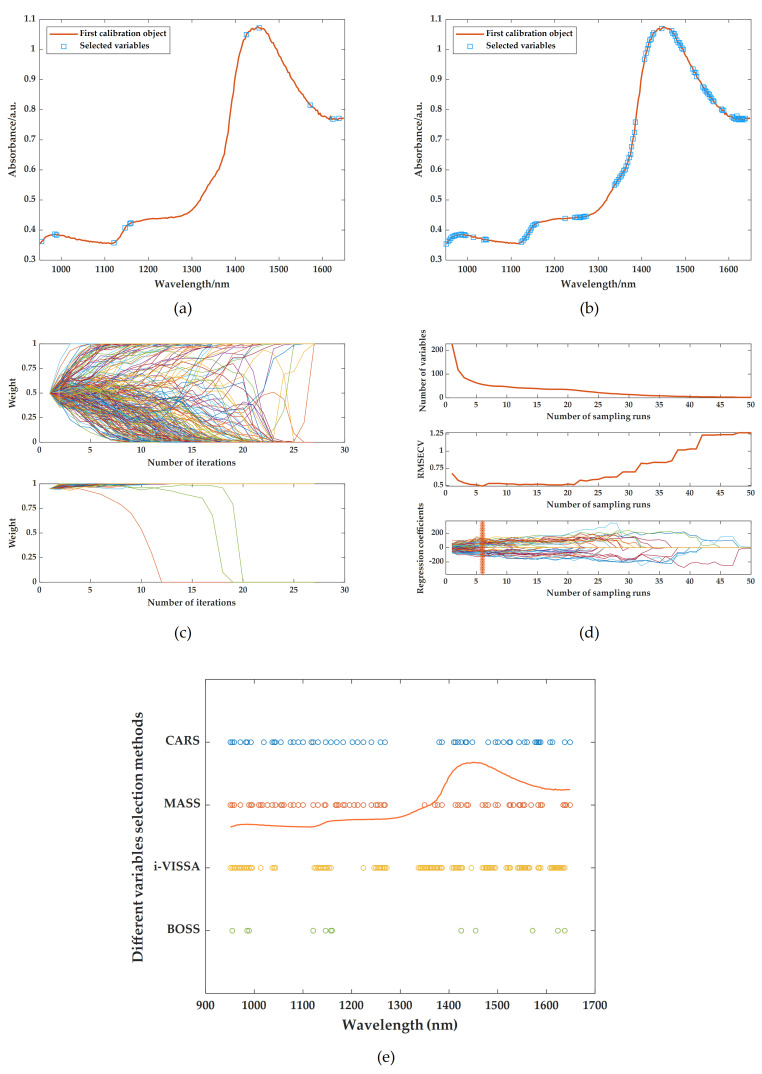
Optimal characteristic wavelengths selected by different variable screening methods: (**a**) extraction results of BOSS after MSC; (**b**) extraction results of i-VISSA after MSC; (**c**) extraction results of MASS after MSC; (**d**) extraction results of CARS after MSC; (**e**) comparison of characteristic wavelength results.

**Figure 8:**
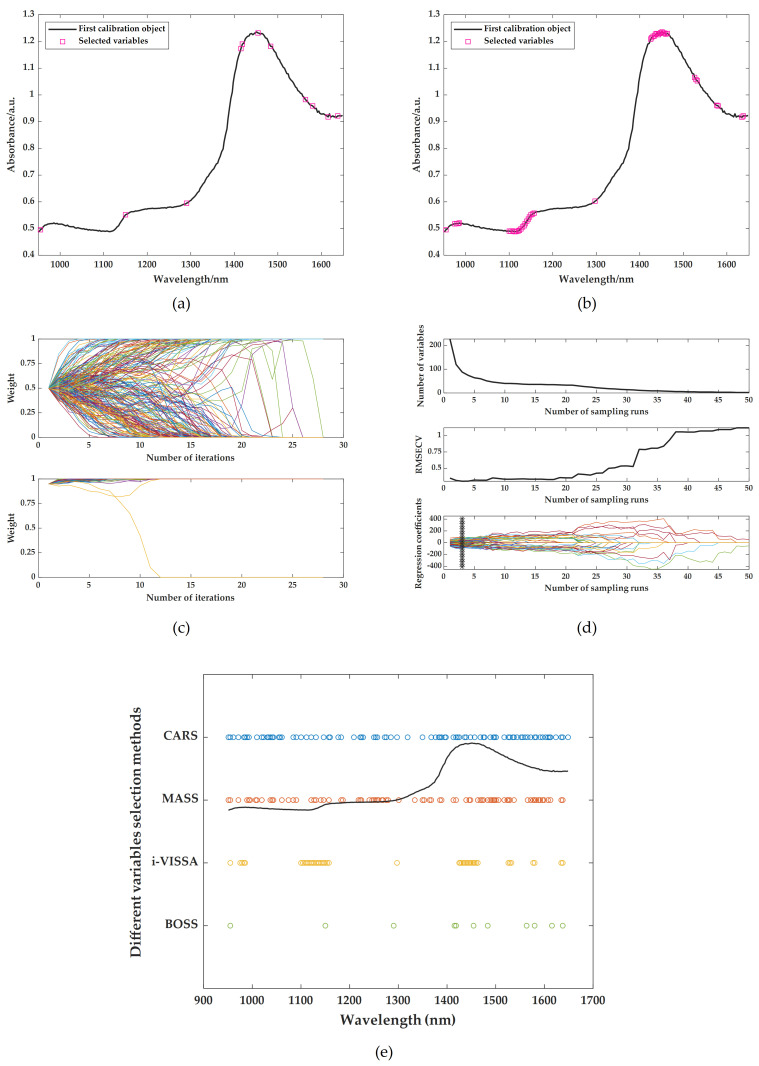
Optimal characteristic wavelengths selected by different variable screening methods: (**a**) extraction results of BOSS after SG; (**b**) extraction results of i-VISSA after SG; (**c**) extraction results of MASS after SG; (**d**) extraction results of CARS after SG; (**e**) comparison of characteristic wavelength results.

Next, based on the above research results, BOSS, CARS, MASS, and i-VISSA are respectively used as variable screening methods to extract spectral characteristic wavelengths. Also, the prediction models are constructed using LSSVR. The screening results of the relevant characteristic wavelengths of TFs, TRs, and TBs are shown in Figure 6a–e, Figure 7a–e and Figure 8a–e respectively. The results of LSSVR models established based on different characteristic wavelengths of the spectrum are shown in Table 5. Among the results of models, only the MASS algorithm played a positive role in the prediction effect. This may be because when the variable screening methods are combined with different preprocessing methods, some combinations may lead to the loss of key spectral feature information, thus affecting the performance of the models. Therefore, it is crucial to select the appropriate combination of spectral pretreatments and characteristic extraction algorithms. The combined approaches of SNV + MASS, MSC + MASS, and SG + MASS are identified as the optimal methods for predicting TFs, TRs, and TBs using spectral information, respectively.

NIRS produces characteristic absorption and reflection peaks by detecting the vibration response of hydrogen-containing groups in the molecular structure of TFs, TRs, and TBs, and then establishes the quantitative relationship model with tea pigments content. However, due to the complex composition of tea, the hydrogen-containing groups of various compounds will produce overlapping absorption in the near-infrared region, which makes the characteristic peaks of tea pigments vulnerable to the interference of coexisting substances, thus limiting the prediction accuracy of the model. In order to improve the predictability and anti-interference ability of the models, the fusion strategies of spectral and image information are proposed.

### 3.6. Prediction Models Based on Multi-Source Information Fusion

In the predictive analysis of comprehensive image and spectral information, the LSSVR model demonstrates superior performance compared to PLSR and SVR. Hence, LSSVR is selected as the modeling method for multi-source information fusion. In low-level data fusion strategy, based on the above experimental results, this study combines the optimal preprocessing methods determined in the image and spectral information modeling stage. Specifically, for different predicted target substances, the best pretreatment schemes that have been verified are respectively adopted for collaborative integration. In terms of middle-level data fusion, the best feature combination extracted from image and spectral information has been further integrated. Feature-level fusion of multi-source data has been achieved. The methods and model results of the fusion of low-level and middle-level data are shown in Table 6.

**Figure 9:**
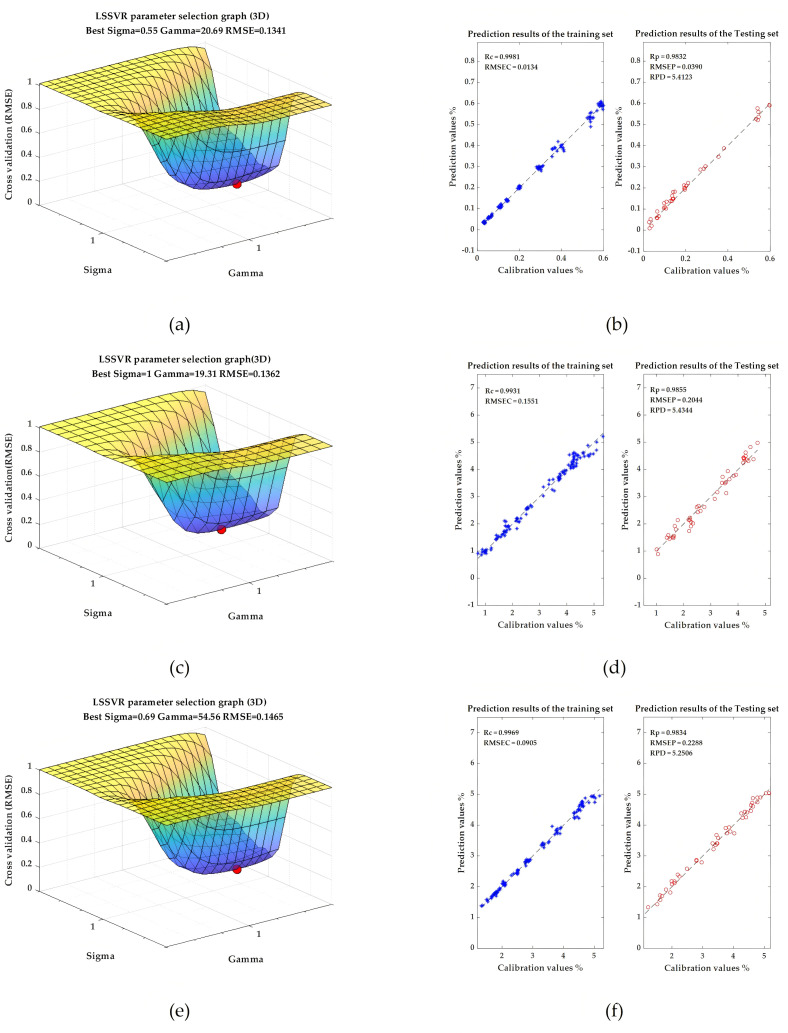
Model optimal results based on LSSVR: (**a**) model parameters optimization for predicting TFs; (**b**) the result of TFs prediction model; (**c**) model parameters optimization for predicting TRs; (**d**) the result of TRs prediction model; (**e**) model parameters optimization for predicting TBs; (**f**) the result of TBs prediction model.

The experimental results show that the results of the prediction models in low-level fusion method are relatively poor, and the effect is less than that of the prediction models established by using the information of the single sensor. This indicates that although the low-level fusion method contains all the data feature information, it also relatively increases redundant information, thereby increasing the complexity of the models. By contrast, by using the middle-level data fusion method, the prediction performance of each model has been effectively improved. The LSSVR model based on the intermediate data fusion strategy has excellent prediction performance for the three target components. The Rp of the testing set of TFs, TRs, and TBs content prediction are 0.9832, 0.9855, and 0.9834, respectively, showing good prediction accuracy. The evaluation indicators of the other results are also quite satisfactory. The prediction results of the best models established for TFs, TRs, and TBs in this study are shown in Figure 9. The results show that middle-level data fusion can effectively fuse the feature information of the two, make the information complementary, eliminate the noise and irrelevant variables between the data, and thereby improve the results of the models.

## 4. Conclusions

In this study, the feasibility of using the fusion information of images and spectral to quantitatively predict the tea pigments content during the rolling process of black tea is discussed. The research shows that although the low-level data fusion method can retain the integrity of multivariate information, it will lead to the expansion of data dimension and the increase of redundant information due to the lack of features extraction and dimension reduction, which will affect the operation efficiency and prediction performance of the model. In contrast, by integrating feature information from different data sources, the middle-level data fusion strategy not only realizes the complementary advantages of multi-source information, but also effectively eliminates redundant noise through feature selection, thus significantly improving the prediction accuracy and robustness of the model. The application of the multi-source information fusion provides the new technological methods for the non-destructive detection of tea pigments during black tea rolling.

## Figures and Tables

**Table 1:** The results of PLSR models for predicting tea pigments based on image information.

Model Selection	Predictive Indicators	Pretreatment Methods	Training Set		Testing Set		
			Rc	RMSEC	Rp	RMSEP	RPD
PLSR	TFs	Z-score	0.9104	0.0897	0.9032	0.1280	1.9682
		Z-score + PCA	0.9240	0.0842	0.9124	0.0818	2.1542
	TRs	Z-score	0.8956	0.5836	0.8828	0.5508	1.9820
		Z-score + PCA	0.9246	0.5055	0.9191	0.5516	2.3890
	TBs	Z-score	0.9161	0.5555	0.9021	0.5111	1.6003
		Z-score + PCA	0.9363	0.4057	0.9289	0.4566	2.2239

**Table 2:** The results of SVR and LSSVR for predicting tea pigments based on image information.

Model Selection	Predictive Indicators	Pretreatment Methods	Training Set		Testing Set		
			Rc	RMSEC	Rp	RMSEP	RPD
SVR	TFs	Z-score + PCA	0.9488	0.0689	0.9314	0.0761	2.4706
	TRs		0.9483	0.4198	0.9404	0.4901	2.4640
	TBs		0.9486	0.3747	0.9352	0.4473	2.4890
LSSVR	TFs		0.9565	0.0863	0.9468	0.0820	2.6070
	TRs		0.9473	0.4015	0.9451	0.4645	2.8988
	TBs		0.9557	0.3635	0.9440	0.4451	2.7910

**Table 3:** The results of PLSR models for predicting tea pigments based on spectral information.

Predictive Indicators	Pretreatment Methods	Training Set		Testing Set		
		Rc	RMSEC	Rp	RMSEP	RPD
TFs	Raw	0.8698	0.1192	0.8179	0.1166	1.4196
	SG	0.8945	0.0966	0.8508	0.1123	1.4780
	MSC	0.8950	0.1031	0.8554	0.1057	1.5802
	SNV	0.9117	0.0868	0.8871	0.1049	1.9764
	Max-min	0.8443	0.1129	0.8374	0.1352	1.4495
	Detrend	0.9065	0.0964	0.8627	0.1155	1.8653
	S-G-1st	0.9036	0.0938	0.8612	0.1397	1.7433
	S-G-2nd	0.8966	0.1046	0.8537	0.1207	1.7210
TRs	Raw	0.9011	0.5743	0.8509	0.6618	1.7021
	SG	0.9151	0.5093	0.8825	0.6263	1.9353
	MSC	0.9357	0.4499	0.9108	0.5486	2.4130
	SNV	0.9042	0.6021	0.8725	0.5911	1.8735
	Max-min	0.8850	0.5865	0.8254	0.7868	1.7433
	Detrend	0.9253	0.5647	0.8800	0.6672	1.9817
	S-G-1st	0.8833	0.6253	0.8341	0.7672	1.6213
	S-G-2nd	0.8925	0.5461	0.8388	0.7619	1.7510
TBs	Raw	0.8750	0.6030	0.8539	0.5898	1.7476
	SG	0.9446	0.4290	0.9326	0.5314	2.2306
	MSC	0.9179	0.4704	0.8822	0.5626	2.0572
	SNV	0.9003	0.5395	0.8817	0.5751	1.6276
	Max-min	0.9082	0.5137	0.8742	0.5665	2.0304
	Detrend	0.9193	0.4951	0.8715	0.6161	1.9551
	S-G-1st	0.9109	0.5039	0.8784	0.5866	2.0458
	S-G-2nd	0.8824	0.5463	0.8779	0.6610	1.6151

**Table 4:** The results of SVR and LSSVR for predicting tea pigments based on spectral information.

Model Selection	Pretreatment Methods	Predictive Indicators	Training Set		Testing Set		
			Rc	RMSEC	Rp	RMSEP	RPD
SVR	SNV	TFs	0.9389	0.0731	0.9186	0.0896	2.2474
	MSC	TRs	0.9450	0.4061	0.9377	0.5144	2.4189
	SG	TBs	0.9498	0.4218	0.9340	0.4356	2.5134
LSSVR	SNV	TFs	0.9475	0.0698	0.9287	0.0799	2.3814
	MSC	TRs	0.9542	0.3776	0.9405	0.4259	2.7297
	SG	TBs	0.9529	0.3565	0.9470	0.4021	2.5627

**Table 5:** The results of LSSVR models established based on different spectral characteristic wavelength selection algorithms.

Predictive Indicators	Pretreatment Methods	Training Set		Testing Set		
		Rc	RMSEC	Rp	RMSEP	RPD
TFs	SNV + BOSS	0.9203	0.0858	0.9117	0.0929	2.0002
	SNV + i-VISSA	0.9414	0.0751	0.9279	0.0862	2.1081
	SNV + MASS	0.9520	0.0685	0.9323	0.0784	2.6718
	SNV + CARS	0.9451	0.0719	0.9289	0.0827	2.3434
TRs	MSC + BOSS	0.9227	0.5121	0.9139	0.5150	2.0690
	MSC + i-VISSA	0.9329	0.4985	0.9216	0.4690	2.2400
	MSC + MASS	0.9605	0.3711	0.9476	0.4100	3.0596
	MSC + CARS	0.9572	0.4338	0.9411	0.4656	2.9377
TBs	SG + BOSS	0.9297	0.4385	0.9160	0.4904	2.2078
	SG + i-VISSA	0.9247	0.4831	0.9177	0.4571	2.0680
	SG + MASS	0.9569	0.3704	0.9476	0.3981	2.8427
	SG + CARS	0.9532	0.3920	0.9266	0.4143	2.6225

**Table 6:** The results of LSSVR models established based on low-level and middle-level data fusion strategies.

Predictive Indicators	Data Fusion Strategy	Pretreatment Methods Combination	Training Set		Testing Set		
			Rc	RMSEC	Rp	RMSEP	RPD
TFs	Low-level	SNV + Z-score	0.9517	0.0650	0.9186	0.0865	2.4280
	Middle-level	SNV + MASS Z-score + PCA	0.9981	0.0134	0.9832	0.0390	5.4123
TRs	Low-level	MSC + Z-score	0.9539	0.3805	0.9360	0.4379	2.6639
	Middle-level	MSC + MASS Z-score + PCA	0.9931	0.1551	0.9855	0.2044	5.4344
TBs	Low-level	SG + Z-score	0.9460	0.3837	0.9160	0.4693	2.3284
	Middle-level	SG + MASS Z-score + PCA	0.9969	0.0905	0.9834	0.2288	5.2506

## Data Availability

The original contributions presented in this study are included in the article. Further inquiries can be directed to the corresponding author.

## References

[1] Li Y., Yu S., Yang S., Ni D., Jiang X., Zhang D., Zhou J., Li C., Yu Z. (2023). Study on taste quality formation and leaf conducting tissue changes in six types of tea during their manufacturing processes. Food Chem.-X.

[2] Ouyang Q., Chang H., Fan Z., Ma S., Chen Q., Liu Z. (2025). Monitoring changes in constituents during black tea fermentation using snapshot multispectral imaging and 1D-CNN enhanced with data augmentation. Comput. Electron. Agric..

[3] Dong H., Li Y., Lai X., Hao M., Sun L., Li Q., Chen R., Li Q., Sun S., Wang B. (2024). Effects of fermentation duration on the flavour quality of large leaf black tea based on metabolomics. Food Chem..

[4] Long P., Kanyasiri R., Ho C., Zhang L. (2023). Thearubigins: Formation, structure, health benefit and sensory property. Trends Food Sci. Technol..

[5] Xu A., Chen L., Shi Y., Wang H., Ye Q., Wang Y., Liu Z., Xu P. (2025). Oxidases and rolling dominate black tea polysaccharide conjugates formation: Enzymatic and non-enzymatic mechanisms. Food Chem..

[6] Huang D., Wang H., Wu Y., Sun C., Fu M., Zhang Y., Yang Y., Wan X., Li Y., Chen Q. (2024). Comprehensive analysis reveals the contribution of “rolling-anaerobic” processing to the improved aroma, taste and color of GABA black tea. Lwt-Food Sci. Technol..

[7] Chen Q., Fu Y., Heng W., Yu S., Xie F., Dong F., Lin Z., Dai W., Fu H. (2024). Re-rolling treatment in the fermentation process improves the taste and liquor color qualities of black tea. Food Chem.-X.

[8] Zhao T., Huang X., Zhao J., Yang C., Zhang S., Huang J., Wang K., Liu Z., Zhu M. (2024). Theaflavins: An underexploited functional compound in black tea. Trends Food Sci. Tech..

[9] Zou H., Shen S., Lan T., Sheng X., Zan J., Jiang Y., Du Q., Yuan H. (2022). Prediction Method of the Moisture Content of Black Tea during Processing Based on the Miniaturized Near-Infrared Spectrometer. Horticulturae.

[10] Zhu J., Zhu X., Yan B., Ren F., Chen B., Han Z., Yao X., He S., Liu H. (2025). Evaluation and categorization of various pea cultivars utilizing near-infrared spectroscopy in conjunction with multivariate statistical techniques. Food Chem..

[11] Zou H., Lan T., Jiang Y., Yu X.-L., Yuan H. (2024). Research on Rapid Detection Methods of Tea Pigments Content During Rolling of Black Tea Based on Machine Vision Technology. Foods.

[12] Gan N., Wang Y., Ren G., Li M., Ning J., Zhang Z., Quan L. (2023). Design and testing of a machine-vision-based air-blow sorting platform for famous tea fresh leaves production. Comput. Electron. Agric..

[13] Liu Z., Zhang R., Yang C., Hu B., Luo X., Li Y., Dong C. (2022). Research on moisture content detection method during green tea processing based on machine vision and near-infrared spectroscopy technology. Spectrochim. Acta Part A Mol. Biomol. Spectrosc..

[14] An T., Jiang Y., Zou H., Xuan X., Zhang J., Yuan H. (2025). Evaluation of Withering Quality of Black Tea Based on Multi-Information Fusion Strategy. Foods.

[15] Xu Q., Zhou Y., Wu L. (2024). Advancing tea detection with artificial intelligence: Strategies, progress, and future prospects. Trends Food Sci. Tech..

[16] Xia Z., Zhou Q., Yang S., Song F., Li Z., Wang J., Ling C., Song C. (2025). Optimization strategy for black tea digital blending by fusing image and spectral information. Food Res. Int..

[17] Wang S., Altaner C., Feng L., Liu P., Song Z., Li L., Gui A., Wang X., Ning J., Zheng P. (2025). A review: Integration of NIRS and chemometric methods for tea quality control-principles, spectral preprocessing methods, machine learning algorithms, research progress, and future directions. Food Res. Int..

[18] Liu H., Chen F., Zhang L., Meng D., Sun H. (2025). Improvement method for tea leaf moisture content prediction using VIS-NIR spectrum based on transfer learning. Spectrochim. Acta Part A Mol. Biomol. Spectrosc..

[19] Shi S., Feng J., Yang L., Xing J., Pan G., Tang J., Wang J., Liu J., Cao C., Jiang Y. (2023). Combination of NIR spectroscopy and algorithms for rapid differentiation between one-year and two-year stored rice. Spectrochim. Acta Part A Mol. Biomol. Spectrosc..

[20] Liu M., Wang R., Shi D., Cao R. (2024). Non-destructive prediction of tea polyphenols during Pu-erh tea fermentation using NIR coupled with chemometrics methods. J. Food Compos. Anal..

[21] Hu Y., Sheng W., Adade S.Y.-S.S., Wang J., Li H., Chen Q. (2025). Comparison of machine learning and deep learning models for detecting quality components of vine tea using smartphone-based portable near-infrared device. Food Control.

[22] Wang Y., Li M., Li L., Ning J., Zhang Z. (2021). Green analytical assay for the quality assessment of tea by using pocket-sized NIR spectrometer. Food Chem..

[23] Liang J., Guo J., Xia H., Ma C., Qiao X. (2025). A black tea quality testing method for scale production using CV and NIRS with TCN for spectral feature extraction. Food Chem..

[24] Wang J., Wang Y., Cheng J., Wang J., Sun X., Sun S., Zhang Z. (2018). Enhanced cross-category models for predicting the total polyphenols, caffeine and free amino acids contents in Chinese tea using NIR spectroscopy. Lwt-Food Sci. Technol..

[25] Chen Y., Gao R., Xiang J., Lin Z., Chen C., Yu W. (2025). Progress of near infrared spectroscopy in tea research. Food Chem..

[26] Chen Y., Guo M., Chen K., Jiang X., Ding Z., Zhang H., Lu M., Qi D., Dong C. (2024). Predictive models for sensory score and physicochemical composition of Yuezhou Longjing tea using near-infrared spectroscopy and data fusion. Talanta.

[27] Yuan L.-M., Mao F., Huang G., Chen X., Wu D., Li S., Zhou X., Jiang Q., Lin D., He R. (2020). Models fused with successive CARS-PLS for measurement of the soluble solids content of Chinese bayberry by vis-NIRS technology. Plant Physiol. Bioch..

[28] Ren G., Yin L., Wu R., Ning J. (2024). Rapid detection of ash content in black tea using a homemade miniature near-infrared spectroscopy. Spectrochim. Acta Part A Mol. Biomol. Spectrosc..

[29] Wen M., Deng B., Cao D., Yun Y., Yang R., Lu H., Liang Y. (2016). The model adaptive space shrinkage (MASS) approach: A new method for simultaneous variable selection and outlier detection based on model population analysis. Analyst.

[30] Zhang H., He Q., Yang C., Lu M., Liu Z., Zhang X., Li X., Dong C. (2023). Research on the Detection Method of Organic Matter in Tea Garden Soil Based on Image Information and Hyperspectral Data Fusion. Sensors.

[31] Li M., Dong S., Cao S., Cui Q., Chen Q., Ning J., Li L. (2023). A rapid aroma quantification method: Colorimetric sensor-coupled multidimensional spectroscopy applied to black tea aroma. Talanta.

[32] He Q., Guo Y., Li X., He Y., Lin Z., Zeng H. (2024). Spectral Fingerprinting of Tencha Processing: Optimising the Detection of Total Free Amino Acid Content in Processing Lines by Hyperspectral Analysis. Foods.

[33] Wang Y., Li L., Liu Y., Cui Q., Ning J., Zhang Z. (2021). Enhanced quality monitoring during black tea processing by the fusion of NIRS and computer vision. J. Food Eng..

[34] Kutsanedzie F., Chen Q., Hassan M., Yang M., Sun H., Rahman M. (2018). Near infrared system coupled chemometric algorithms for enumeration of total fungi count in cocoa beans neat solution. Food Chem..

